# Analysis of factors determining thermal changes at osteotomy site in dental implant placement - An *in-vitro* study

**DOI:** 10.4317/jced.57346

**Published:** 2021-03-01

**Authors:** Radhu Raj, V Manju, Vinod Kumar-Gopal, Manu Eswar

**Affiliations:** 1 Assistant Professor, Department of Prosthodontics and Crown and Bridge. Amrita School of Dentistry. Amrita Institute of Medical Sciences and Research Centre. Amrita Vishwa Vidyapeetham University, Kochi, Kerala, India; 2 Professor, Department of Prosthodontics and Crown and Bridge. Amrita School of Dentistry. Amrita Institute of Medical Sciences and Research Centre. Amrita Vishwa Vidyapeetham University, Kochi, Kerala, India; 3 Assistant Professor, Department of Mechanical Engineering, Amritapuri Campus, Kollam, Kerala, India; 4 Staff Engineer, Amrita Centre for Nanosciences, Amrita Institute of Medical Sciences and Research Centre. Amrita University, Kochi, Kerala, India

## Abstract

**Background:**

Heat generation during osteotomy site preparation is a crucial factor that determines the success of dental implant placement. Among the factors that affect the heat generation, drilling speed, hand pressure and coolant temperature are independent variables. However, a relation between these three parameters and their optimal values required for the maximum outcome has not been studied so far. This study aims at finding out a relation between these factors in order to derive the optimum balance required, using an *in vitro* study.

**Material and Methods:**

This *in vitro* experiment was performed on bovine femur. A total of 72 drillings were undertaken with the aid of a physiodispenser mounted on the test apparatus. Drill diameters of 2 mm and 2.8 mm, rotated at 1500, 2000 and 2500 rpm were included for the analysis. Hand pressures included for the comparison were 1.2 kgf and 2.4 kgf. Normal saline at room temperature, and that chilled to 00C were used for external irrigation. The temperature generated during drilling was recorded by infrared thermography using a Forward-Looking Infrared (FLIR) camera.

**Results:**

The highest temperature during osteotomy was observed at 2000 rpm rotational speed, 1.2 kgf operator hand pressure and saline irrigant solution at room temperature. In contrast, the lowest temperature generated was using 2500 rpm rotational speed, 2.4 kgf operator hand pressure and chilled irrigant solution.

**Conclusions:**

The results indicate that none of the three experimented parameters generated heat above the critical temperature for bone necrosis (47°C). Thus, a high drilling speed with high hand pressure and continuous irrigation with copious amounts of cooled saline may be the ideal combination for implant osteotomy site preparation.

** Key words:**Heat generation, dental implant drills, drilling speed, drilling pressure, irrigation, infrared thermography, thermal necrosis, osteotomy preparation.

## Introduction

Dental implants offer an excellent option to address the limitations of conventional dentures, bridges and missing teeth. Placement of an implant involves drilling a hole in the jaw bone, and threading the implant into the prepared hole. This site is known as the osteotomy site. Over time, bone gets deposited on the implant surface, a phenomenon known as osseointegration which is critical for a high implant success rate of up to 15 years (1).

There are many parameters that must be considered during implant placement for adequate osseointegration to occur because failure of proper osseointegration jeopardizes the stability of the implants. This can be early failure (due to non-osseointegration), or delayed failure (2). Early failure is attributed to one or more problems during the osteotomy site preparation. One such problem is overheating of the bone due to friction during the drilling process. Previous studies have demonstrated that healing of the osseous structure occurs either by repair or regeneration, and the progression of bone healing dictates the outcome of the implant. Also, thermal damage to the bone and subsequent necrosis is proportional to the combined result of the heat generated during drilling and the period of time the bone tissue is at the elevated temperature (3).

As bone has a poor thermal conductivity (0.38 ± 23 J/msK), the thermal necrosis that ensues due to the undissipated heat generated could compromise the primary stability of the dental implant (4). This thermal injury can result in fibrous tissue formation at the implant-bone interface, compromising the long-term prognosis (5). Literatures indicate that temperatures ranging from 56 to 700C are harmful to bone tissue because of the denaturation of stored alkaline phosphatase (AP) in the bone. Also, it is known that functional bone regeneration occurs when a temperature of 470C is not exceeded for one minute (6).

Various techniques have been recommended to reduce the amount of frictional heat generated during osseous preparation, and there are various results published about the optimal speed required for drilling. Also, drill speed is not an independent factor in heat production, but it is associated with hand pressure (7). Moreover, use of coolants is a highly important factor for preserving the vitality of bone, reducing friction and facilitating bone chip removal (8).

So far, there has been limited evidence that relates the common clinical factors like the drilling speed, the amount of pressure the operator applies to the hand-piece, the temperature of the irrigant used and the resultant frictional heat that is generated. Therefore, a detailed in-vitro experimental study on bovine bone was been performed in combination with the above mentioned parameters.

## Material and Methods

An *in vitro* study was performed in accordance with the accepted standards, and the study was approved by the Dissertation Review Committee of Amrita School of Dentistry, Amrita Institute of Medical Sciences and Research Centre, Kochi, Kerala, India.

The bone chosen for the study was bovine femur because of its structural resemblance to human bone, while also having a constant cortical thickness (9,10). They were cut into 12 cm long samples, and stored in Neutral Buffered Formalin (NBF) solution.

-Test apparatus and experimental set-up

**Figure 1:**
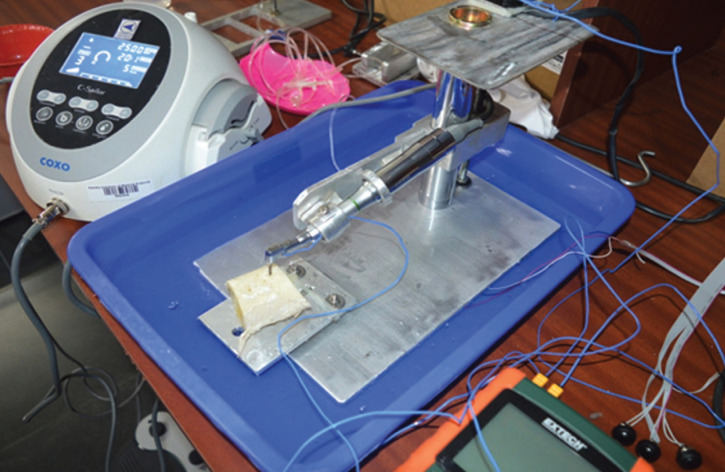
Test apparatus.

The major components included a drilling machine (COXO Dental Physiodispenser, INDIA), two drill bits (ADIN Dental Implant Systems Ltd., Israel), a few bovine bone blocks, a load cell, a burette and a drill jig (Fig. 1).

The automated drill jig composed of two main entities - the drill head, which feeds the handpiece at a constant feed rate (0.9mm/s) into the bone specimen and a rigid bed on to which a vice holds the bone specimen. The bone specimens were secured in a custom-made screw-assisted metal holder attached to the device. The load on the vice is instantaneously monitored with the help of a load cell so as to maintain the drill force under limits of 1.2 and 2.4kgf. The load cell was calibrated to three points with two known masses. The errors and deviation (non-linearity) were less than 1%. All drilling procedures were done to a uniform depth of 10 mm with a constant torque (35 Ncm) and a constant feed rate (0.9 mm/s). Two 13 mm long new implant twist drills of diameters 2 and 2.8mm were used. Thirty six osteotomy sites were prepared using the physiodispenser mounted on the test apparatus.

**Figure 2:**
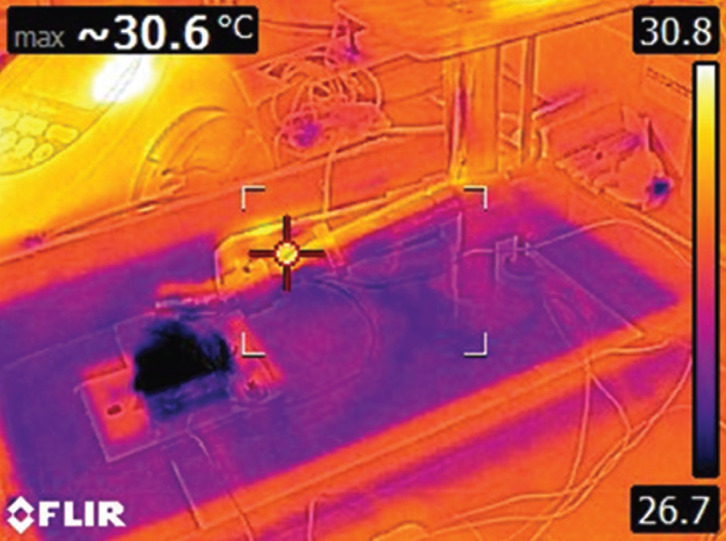
Thermographic image.

At the osteotomy site, the first drilling was performed with the 2 mm drill bit and then graduated with the 2.8 mm drill bit. Three different rotational speeds i.e. 1500, 2000 and 2500 rpm were compared. Hand pressures of 1.2 kgf and 2.4 kgf were calibrated in the test apparatus to mimic the operator’s hand pressure. Drill sites were randomly assigned. Normal saline solution (Fresenius Kabi, Pvt Ltd, India) at room temperature, and that chilled to 00C were used to irrigate the site, and its flow was maintained throughout drilling at a rate of 40 mL/min. The irrigant solution was fed directly to the drilling site through a burette from the temperature controlled bath to simulate the clinical scenario. The irrigant feed rate was measured by observing the time required to empty the burette. Each bone specimen was used for up to 5 osteotomies. Thermal measurements were performed in a climate-controlled room (temperature ~ 280C). The maximum observed change in temperature from baseline, delta t max 0C (Ʌt) was examined as the principal factor that represented the thermal insult to the bone. Thermographic images obtained using the infrared (IR) camera (FLIR TG165, U.S) enabled the estimation of rise in temperature (Fig. 2). In these obtained thermographic images, each temperature isotherm was denoted with a different colour, which enabled an excellent overview of the temperature distribution. Table 1 lists the various groups used for the study (n=5 for each group). Figure 1 represents the experimental setup – test apparatus.

**Table 1:**
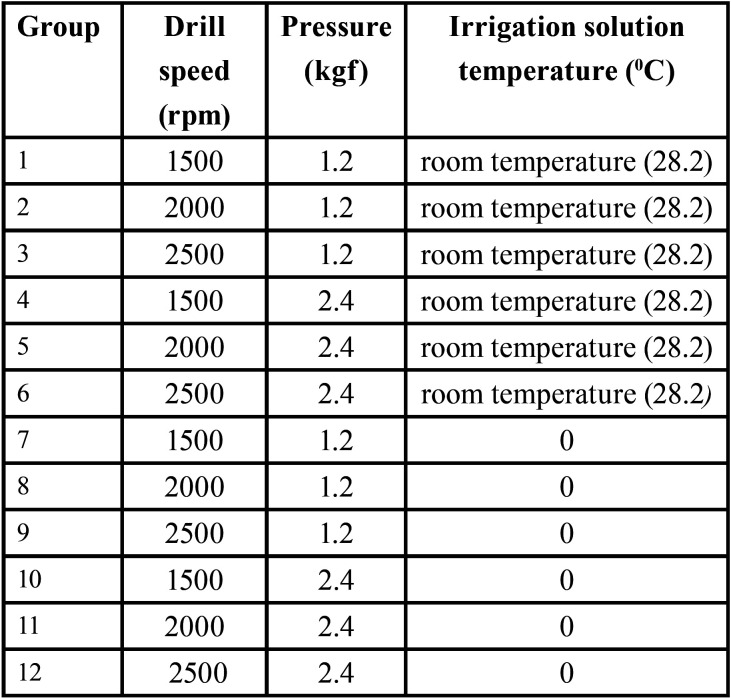
Each study group shows a combination of varied parameters of drilling speed, operator hand pressure and irrigant solution temperature.

-Statistical analysis

The data was expressed as mean ± standard deviation (S.D.). F-test was used to compare temperature changes at different drilling speeds with respect to different drill diameters. The T-test was used to compare the temperature change at varying pressures and irrigant temperature with respect to drill diameters. The Kruskal-Wallis test, a one-way ANOVA test, was used to compare the temperature changes among the study groups for drill diameters 2mm and 2.8 mm. Further, Mann-Whitney U-test was used to compare the temperature changes between the study groups. A *p*-value < 0.05 was considered statistically significant.

## Results

-Drilling speed

**Figure 3:**
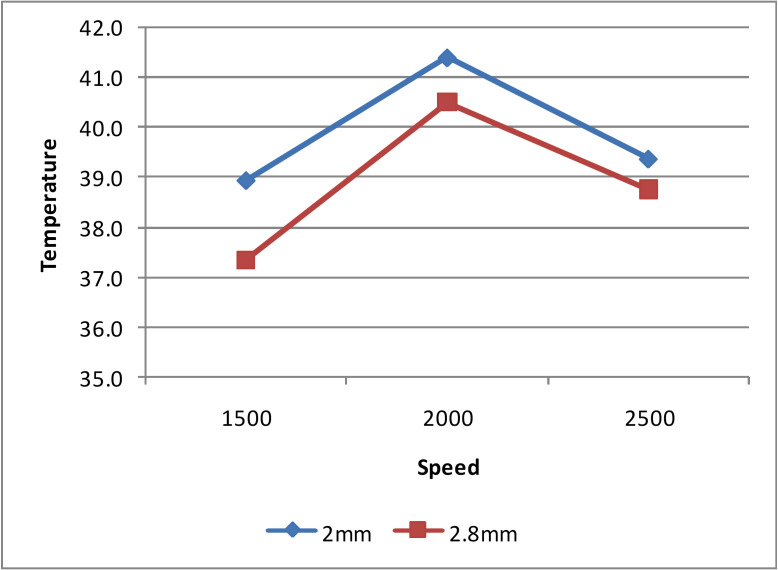
Thermal changes at osteotomy site with varying drill speed and drill diameter.

The effect of drilling speed and drill diameter on temperature rise was measured (Fig. 3). The temperature change observed was 38.9±4.50C for 1500 rpm; 41.1± 5.70C for 2000 rpm and 39.4± 4.30C for 2500 rpm drill speed with 2mm drill, and 37.3± 4.20C for 1500 rpm, 40.5±5.60C for 2000 rpm and 38.8±3.80C for 2500 rpm drill speed with 2.8 mm drill diameters. Although f – test did not show any significant statistical difference (*p* > 0.01), the mean ± S.D values suggested that out of the three drilling speeds assessed, 2000 rpm generated slightly more heat when compared to the drilling speeds 1500 rpm and 2500 rpm irrespective of the drill diameter.

-Drilling pressure

The effect of operator hand pressure and drill diameter on temperature rise was measured. The temperature observed was 41.0± 4.40C for 1.2 kgf, 38.8 ± 5.20C for 2.4 kgf hand pressure with the 2 mm drill, 39.7 ± 4.20C for 1.2 kgf, and 38.1± 5.00C for 2.4 kgf hand pressure with the 2.8 mm drill. There was no statistically significant rise in temperature while varying the drilling pressure. However, there was a significant decrease in temperature generated while using a relatively high hand pressure of 2.4kgf when compared to 1.2 kgf on comparing the independent study groups such as 1,2,3 with 4,5,6, and 7,8,9 with 10,11 and 12.

-Irrigant solution temperature

**Figure 4:**
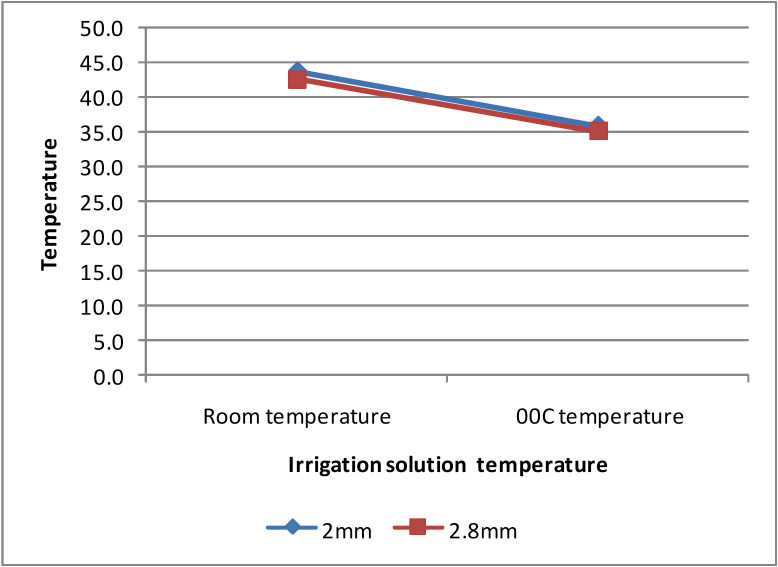
Thermal change at osteotomy site with varying irrigant solution temperature and drill diameter.

The effect of irrigant solution temperature and drill diameter on temperature rise was measured (Fig. 4). The temperature change for irrigant solution at room temperature was 43.9±2.60C for 2mm drill, and 42.6±2.70C for 2.8 mm drill. The temperature change for chilled irrigant solution was 36.0±3.00C for the 2 mm drill, and 35.1±2.70C for 2.8 mm drill. From the results obtained, the chilled irrigant solution showed a statistically significant (*p*<0.01) decrease in temperature compared to the irrigant solution at room temperature.

The effect of temperature rise for each study group of drill diameters 2 mm and 2.8 mm were recorded. The individual parameters considered for each study group is given in Table 1. Group 2 (2000 rpm speed, 1.2kgf pressure, irrigant solution at room temperature ~ 28.2 0C) showed a relatively high temperature generation followed by groups 7, 8 and 9 irrespective of the drill diameter. While comparing group 2 with group 5, the drilling speed and irrigant solution temperature were the same, but there was a decrease in temperature generated in group 5 in which the drilling was performed with 2.4 kgf hand pressure. Similarly, the heat generated in groups 10, 11 and 12 was significantly reduced in comparison to groups 7, 8 and 9. In addition, the comparison of groups 10, 11 and 12 with other study groups under consideration suggested that there was a significant decrease (*p*< 0.01) in temperature generated on using chilled irrigant solution than with irrigant solution at room temperature.

## Discussion

Heat generated during osteotomy site preparation and excessive operator hand pressure at the crestal region leads to implant bone loss during the healing period. A few case reports have described that implant failure could be due to bone overheating, which induces thermal necrosis. In these, the histological features of the specimens were presented and proposed that the most probable cause of failure was bone overheating although other causes could not be excluded (11). A case report regarding implant-related periapical lesions suggested a combination of bone overheating and bone chip compression during implant placement as the reason for implant failure (12). The authors indicated that implant failures may be caused by a combination of multiple factors, and since it is not always possible to accurately control each factor, as can be done in *in vivo* studies, it is difficult to determine the prime etiological factor in a clinical scenario. Thus, although the exact cause of implant failure in these case reports remains undetermined, thermally induced bone necrosis may be the most likely cause of implant failure. From our study, we understand that preparing an implant osteotomy site at 2500 rpm could decrease the risk of osseous damage thereby providing a favorable milieu for initial bone integration. Hence, avoiding the development of a devital zone adjacent to the implant facilitates better immediate load bearing on the implant.

Regarding the drilling speed, certain implant systems recommend drilling speeds ranging from 800 to 1500 rpm in order to avoid overheating (13). But, this means that with a lower drilling speed the drilling time is also prolonged, requiring more time for the bone to return to the baseline normal temperature. In another study, a reduction in drilling time up to 30 to 40% was observed using 2,400 rpm speed with 2,400g force (14). The concept of low-speed drilling has been suggested as an alternative to the conventional procedure which has pros such as easy manipulation of the drilling path, and cons such as a longer drilling time. It is reported that the strength of low-speed drilling can be easily controlled to maintain the path of drilling. Hence, conventional drilling can change its drilling path on its own when the drill encounters a dense cortical bone (13). From the results obtained, although no significant difference among the three drilling speeds were observed with respect to temperature generation, 2,000 rpm generated more heat compared to 1,500 and 2,500 rpm. Regarding 1500 rpm, previous reports have indicated that lower speed such as 1500 rpm requires longer drilling time which may hamper bone healing leading to bone necrosis. Higher speeds (2,500 rpm), will reduce the drilling time as compared to lower speeds, and generate less heat that can return back to baseline temperature in a shorter time. Clinical trials by BioHorizons® Dental Implants that used a drill speed of 2,500 rpm during a 3-year period reported 99% survival with well integrated implants in all bone densities (7). The results of our study also recommend 2,500 rpm as the ideal drilling speed. However, further *in vivo* studies are mandatory.

Increasing both the speed and the drilling pressure together allowed for more efficient cutting with no significant increase in temperature (14). It was also reported that increasing the rate of advancement of the drill by increasing the drilling force does not increase heat production. However, lesser load actually resulted in greater heat production (15). It was demonstrated that the average force placed on the hand piece during osseous preparation is 1.2 kg, but its influence on heat generation wasn’t investigated (16). From the results obtained, no statistically significant increase in temperature with varying drilling pressure was observed. However, on analyzing the independent study groups, a significant difference in temperature generated was observed using a relatively high hand pressure of 2.4 kgf compared to the lower pressure of 1.2 kgf. Hence, until proven otherwise, hand pressure that usually falls in the range of 2 kgf should be applied throughout the osteotomy procedure to minimize heat generation.

In order to avoid thermal damage to the bone, low drilling speeds and simultaneous irrigation during drilling (with sterile physiological saline) is conventionally recommended (17). In conditions of osteotomy preparation without irrigation, drill temperatures exceed 47°C within a few seconds. Prompt irrigation greatly aids in lowering drill temperature. Clogged drill flutes decrease its cutting efficiency and blocks irrigation, thus contributing to a rise in drill temperature (18).

In our study, the effect of the irrigant solution temperature and drill diameter on temperature rise were measured, which showed lesser heat generation on the use of chilled irrigant solution compared to the irrigant solution at room temperature. This could be attributed to the use of a higher drill pressure of 2.4 kgf with a constant irrigant temperature and drill speed. None of the above drilling parameters generated heat exceeding 470C, which is the critical temperature for bone necrosis.

## Conclusions

Although no significant difference in heat generation at varying drill speeds and operator hand pressures was observed, none of the above drilling parameters generated heat exceeding 470C which is the crucial temperature for bone necrosis. Thus, a high drilling speed of 2500 rpm with a high hand pressure while drilling and continuous irrigation with copious amounts of cooled saline irrigation solution seems to be the ideal for implant osteotomy site preparation in order to minimize heat generation.
